# A Proteomic Approach to Investigating Gene Cluster Expression and Secondary Metabolite Functionality in *Aspergillus fumigatus*

**DOI:** 10.1371/journal.pone.0106942

**Published:** 2014-09-08

**Authors:** Rebecca A. Owens, Stephen Hammel, Kevin J. Sheridan, Gary W. Jones, Sean Doyle

**Affiliations:** Department of Biology, National University of Ireland Maynooth, Maynooth, Co. Kildare, Ireland; Woosuk University, Republic of Korea

## Abstract

A combined proteomics and metabolomics approach was utilised to advance the identification and characterisation of secondary metabolites in *Aspergillus fumigatus*. Here, implementation of a shotgun proteomic strategy led to the identification of non-redundant mycelial proteins (*n* = 414) from *A. fumigatus* including proteins typically under-represented in 2-D proteome maps: proteins with multiple transmembrane regions, hydrophobic proteins and proteins with extremes of molecular mass and p*I*. Indirect identification of secondary metabolite cluster expression was also achieved, with proteins (*n* = 18) from LaeA-regulated clusters detected, including GliT encoded within the gliotoxin biosynthetic cluster. Biochemical analysis then revealed that gliotoxin significantly attenuates H_2_O_2_-induced oxidative stress in *A. fumigatus* (*p*>0.0001), confirming observations from proteomics data. A complementary 2-D/LC-MS/MS approach further elucidated significantly increased abundance (*p*<0.05) of proliferating cell nuclear antigen (PCNA), NADH-quinone oxidoreductase and the gliotoxin oxidoreductase GliT, along with significantly attenuated abundance (*p*<0.05) of a heat shock protein, an oxidative stress protein and an autolysis-associated chitinase, when gliotoxin and H_2_O_2_ were present, compared to H_2_O_2_ alone. Moreover, gliotoxin exposure significantly reduced the abundance of selected proteins (*p*<0.05) involved in *de novo* purine biosynthesis. Significantly elevated abundance (*p*<0.05) of a key enzyme, xanthine-guanine phosphoribosyl transferase Xpt1, utilised in purine salvage, was observed in the presence of H_2_O_2_ and gliotoxin. This work provides new insights into the *A. fumigatus* proteome and experimental strategies, plus mechanistic data pertaining to gliotoxin functionality in the organism.

## Introduction

Following the publication of *A. fumigatus* Af293 [1] genomic sequence and the sequencing of a second *A. fumigatus* strain, A1163 [2], extensive efforts have been undertaken to characterise the proteome of this opportunistic human pathogen [3]–[10]. Traditional proteomic strategies have utilised 2-D separation with subsequent protein identification by MS. Shotgun MS-based proteomics has developed more recently and provides a complementary method to 2-D for proteome profiling [9], [10], since 2-D can occasionally be limiting for the identification of particular subsets of proteins, especially hydrophobic proteins, membrane proteins, and proteins with large molecular mass or extreme p*I* [11].

MS-based or shotgun proteomics can adopt multiple approaches including, (i) direct LC-MS/MS, (ii) indirect LC-MS/MS and (iii) 2-D-LC-MS/MS (multidimensional protein identification technology, MudPIT) [12], [13]. Direct LC-MS/MS involves the on-line separation of complex peptide mixtures using reversed phase nano-LC columns with extended acetonitrile gradients to effect peptide separation [14]. Indirect LC-MS/MS is where complex peptide or protein mixtures are pre-fractionated off-line (e.g. by SDS-PAGE) before LC-MS/MS analysis [15]. Sub-proteome strategies have also been implemented to investigate glutathione binding [4] and mitochondrial proteins [6]. Indeed, the recent emergence of MS-based proteomics studies of *A. fumigatus* has been undertaken whereby 530 plasma membrane associated proteins were identified by utilising a combination of SDS-PAGE fractionation of total protein followed by peptide separation and identification by 2-D-LC-MS/MS [16]. This study would have been difficult to perform using 2-D due to the incompatibility of hydrophobic proteins, and proteins with transmembrane (TM) regions, with detergents used in isoelectric focusing, the first separation stage of 2-D [17]. Quantitative MS-based proteomics, both label-free and using isobaric tagging for relative and absolute quantitation (iTRAQ), have been used to comparatively profile the stages of *A. fumigatus* germination [9], [10]. Activity-based MS proteomics has also recently been developed to investigate *A. fumigatus* following incubation with human sera [18]. The application of MS-based proteomics to dissect the proteome of *A. fumigatus* has the potential to provide a global overview of the pathways and biological processes active under a set of conditions. In addition, (i) bioinformatic analysis can expand the characterisation of large datasets generated by MS-based proteomics, and (ii) shotgun proteomics offers the possibility of identifying the presence of either hypothetical proteins or proteins of unknown function, whose existence may either be unclear, or only previously demonstrated at the transcript level. Furthermore, (iii) shotgun MS-based proteomics has the potential to be used for the non-targeted identification of secondary metabolite (SM) cluster expression, which, coupled with subsequent metabolomics, could result in the identification of novel cluster products [19].

Proteomic approaches may also have an application in characterizing the effect of exogenous SMs on *A. fumigatus* [20]. Indeed, despite the many advantages of shotgun proteomics, 2-D has been successfully deployed to inform on proteomic alterations in *A. fumigatus* under various conditions [20]–[27]. Thus, a complementary strategy of shotgun and 2-D proteomics offers much in terms of the ability to reveal the nature of the proteome in pathogenic microorganisms, provide further insight into SM biosynthesis- and explore how apparently synergistic stressors may interact in unexpected ways. Interestingly, both gliotoxin and H_2_O_2_, separately, have been shown to result in numerous, growth inhibitory-associated, alterations to the proteome of *A. fumigatus* [20], [23], [28]. Indeed, exposure of mammalian cells to gliotoxin has been shown to increase the production of ROS, while H_2_O_2_ induces oxidative stress [29]. Paradoxically however, it has been revealed [30] that gliotoxin actually relieved H_2_O_2_–induced growth inhibition of *A. fumigatus* in a concentration-dependent manner, although the basis for this phenomenon was not investigated.

The aim of the work presented here was to investigate the potential for shotgun MS to dissect the mycelial proteome, particularly with respect to identifying SM cluster expression, allied to cognate metabolite biosynthesis. Moreover, dissection of the molecular basis of SM (gliotoxin)-mediated relief of H_2_O_2_-induced oxidative stress in *A. fumigatus* was explored by 2-D and LC-MS/MS analysis. Overall, these combinatorial approaches reveal new insights into the expression, functionality and dynamic nature of the *A. fumigatus* proteome during normal growth and consequent to attenuated oxidative stress conditions.

## Materials and Methods

### Mycelial proteomics

For shotgun proteomics, mycelia from *A. fumigatus* ATCC26933 shaking cultures were harvested after 48 h, 200 rpm, 37°C in *Aspergillus* minimal media (AMM) and snap-frozen in liquid nitrogen. *A. fumigatus* ATCC26933 mycelia were also harvested from shaking cultures grown for 72 h in Czapek-Dox media, 37°C, 200 rpm. Protein was extracted and subjected to trypsin digestion as described [31]. Briefly, frozen mycelia (1 g) were ground in liquid nitrogen and resuspended in 6 ml of 25 mM Tris-HCl, 6 M Guanidine-HCl, 10 mM DTT pH 8.6. Extracts were sonicated five times at 10% power, cycle 6 for 10 sec intervals, followed by centrifugation at 10000 g for 10 min at 4°C. DTT (1 M; 10 µl per ml lysate) was added to the supernatants and incubated at 56°C for 30 min. Iodoacetamide (1 M; 55 µl per ml lysate) was added and incubated in the dark for 20 min. Whole cell lysates were dialysed twice against 100 mM ammonium bicarbonate. Aliquots of denatured protein solutions (100 µl) were digested with trypsin (5 µl; 0.4 µg/µl in 10% (v/v) acetonitrile, 10 mM ammonium bicarbonate), overnight at 37°C. Tryptic peptide mixtures were spin-filtered (Agilent Technologies, 0.22 µm cellulose acetate), separated on extended liquid chromatography gradients on a nanoflow Agilent 1200 LC system and subjected to tandem mass spectrometry using an Agilent 6340 Ion Trap LC-MS System (Agilent Technologies, Santa Clara, CA). Database searches for identification of proteins were carried out using Spectrum Mill MS Proteomics Workbench (Revision B.04.00.127). Validation criteria were set to (i) maximum of two missed cleavages by trypsin, (ii) fixed modification: carbamidomethylation of cysteines, (iii) variable modifications: oxidation of methionine, (iv) mass tolerance of precursor ions ±2.5 Da and product ions ±0.7 Da were employed and searches were carried out against a protein database of *Aspergillus fumigatus* strains Af293 (reference strain) and A1163, acquired from [32]. Protein grouping was carried out based on the presence of ≥1 shared peptide. Protein identifications were validated based on fixed thresholds (minimum protein score set to 20), with single peptide identifications requiring a Spectrum Mill score ≥17.0 and SPI >70%. In order to determine the relative hydrophobicity of the identified proteins, the grand average of hydropathy (GRAVY) index was calculated using GRAVY calculator (www.gravy-calculator.de). Using Phobius (http://phobius.cbr.su.se), the number of putative transmembrane regions present in each identified protein was determined. Identified proteins were grouped into functional categories based on the FunCat (Functional Catalogue), GO (Gene Ontology) and KEGG (Kyoto Encyclopedia of Genes and Genomes) annotations, using the FungiFun application (https://www.omnifung.hki-jena.de/FungiFun/) [33].

### Detection of Secreted Secondary Metabolites in *A. fumigatus*

Culture supernatants were harvested from *A. fumigatus* ATCC26933 grown for 48 h in AMM or 72 h in Czapek-Dox, as described above. Organic extractions were carried out using a 1∶1 mixture of chloroform to culture supernatant [34]. Chloroform extracts were dried to completion using a rotary evaporator and resuspended in methanol for LC-MS analysis using an Agilent 6340 Ion Trap LC-MS System (Agilent Technologies, Santa Clara, CA). Settings were adjusted to include the detection of singly charged molecules and molecules were separated on an acetonitrile gradient over a 15 min runtime.

### 2-D and LC-MS/MS

The mechanisms involved in gliotoxin-mediated relief of H_2_O_2_-induced stress in *A. fumigatus* ATCC26933 were investigated by comparative 2-D and LC-MS/MS. *A. fumigatus* ATCC26933 was grown in Sabouraud dextrose media at 200 rpm, 37°C for 24 h prior to addition of one of the following four treatments: (i) Solvent control (500 µl methanol added per 50 ml culture), (ii) Gliotoxin alone (gliotoxin, dissolved in methanol, added to a final concentration of 10 µg/ml), (iii) H_2_O_2_ alone (H_2_O_2_ added to a final concentration of 2 mM; 500 µl methanol added per 50 ml culture), (iv) Gliotoxin and H_2_O_2_ combined (gliotoxin added to a final 10 µg/ml and H_2_O_2_ added to final 2 mM). Mycelia (*n* = 5 biological replicates/treatment) were harvested after 4 h and ground in liquid nitrogen. Crushed mycelia were resuspended in 10% (w/v) TCA and sonicated three times at 10% power, cycle 6, 10 sec. Samples were incubated on ice for 20 min and centrifuged at 10000 g, 4°C for 10 min. Pellets were washed with ice-cold acetone to remove excess TCA and resuspended in IEF buffer [4]. Protein was separated on pH 4–7 IEF strips, followed by resolution by SDS-PAGE [35], [36]. Colloidal Coomassie staining was carried out on gels, with subsequent protein spot analysis using Progenesis SameSpot software (Nonlinear Dynamics, Newcastle upon Tyne, United Kingdom). Gels (*n* = 20; 5 replicates/treatment) from all four treatments were aligned and subsets of treatments were compared. Spots demonstrating significant changes in abundance (*p*<0.05, fold change ≥1.5) were excised from gels and trypsin digested according to the method described by Shevchenko (2007) [37]. Digested peptides were analysed by LC-MS/MS as described previously, with SpectrumMill used for database searching.

### Fluorescent Detection of ROS in *A. fumigatus*

*A. fumigatus* ATCC26933 conidia (4×10^6^ per well, in 6 well plates) were added to Sabouraud liquid media (4 ml/well) and incubated for 24 h at 37°C, static. Each well contained a glass microscope slide. After removal of the top layer of mycelia, H_2_O_2_ (2 mM final) only, or with gliotoxin (10 µg/ml final) was added. H_2_O_2_ alone acts as a positive control as it is a known ROS inducing agent. Gliotoxin (10 µg/ml final) and an equivalent volume of MeOH were also added individually to separate wells as negative control. Plates were re-incubated 37°C, static, for 30 min, culture supernatants removed and mycelial mats washed once with 4 ml PBS for 5 min. After PBS removal, Sabouraud medium (3 ml) containing 2′,7′-dichlorodihydrofluorescein diacetate (2.5 µg/ml; H_2_DCFDA; Life Technologies) was added to each well followed by incubation 37°C for 40 min, static. After washing twice with PBS (2×20 min each), mycelia were visualised using a fluorescent microscope (GFP filter: Ex/Em: 492–495/517–527 nm). Fluorescence was quantified by measuring Integrated Density Value (IDV) of selected areas from each image (*n* = 5/treatment) representing subsequent data as means ± standard error. Differences were evaluated using ANOVA, and statistical significance was accepted at *p*<0.05.

## Results

### Shotgun mass spectrometry identifies 414 proteins from *A. fumigatus* mycelia grown in AMM

**Figure 1:**
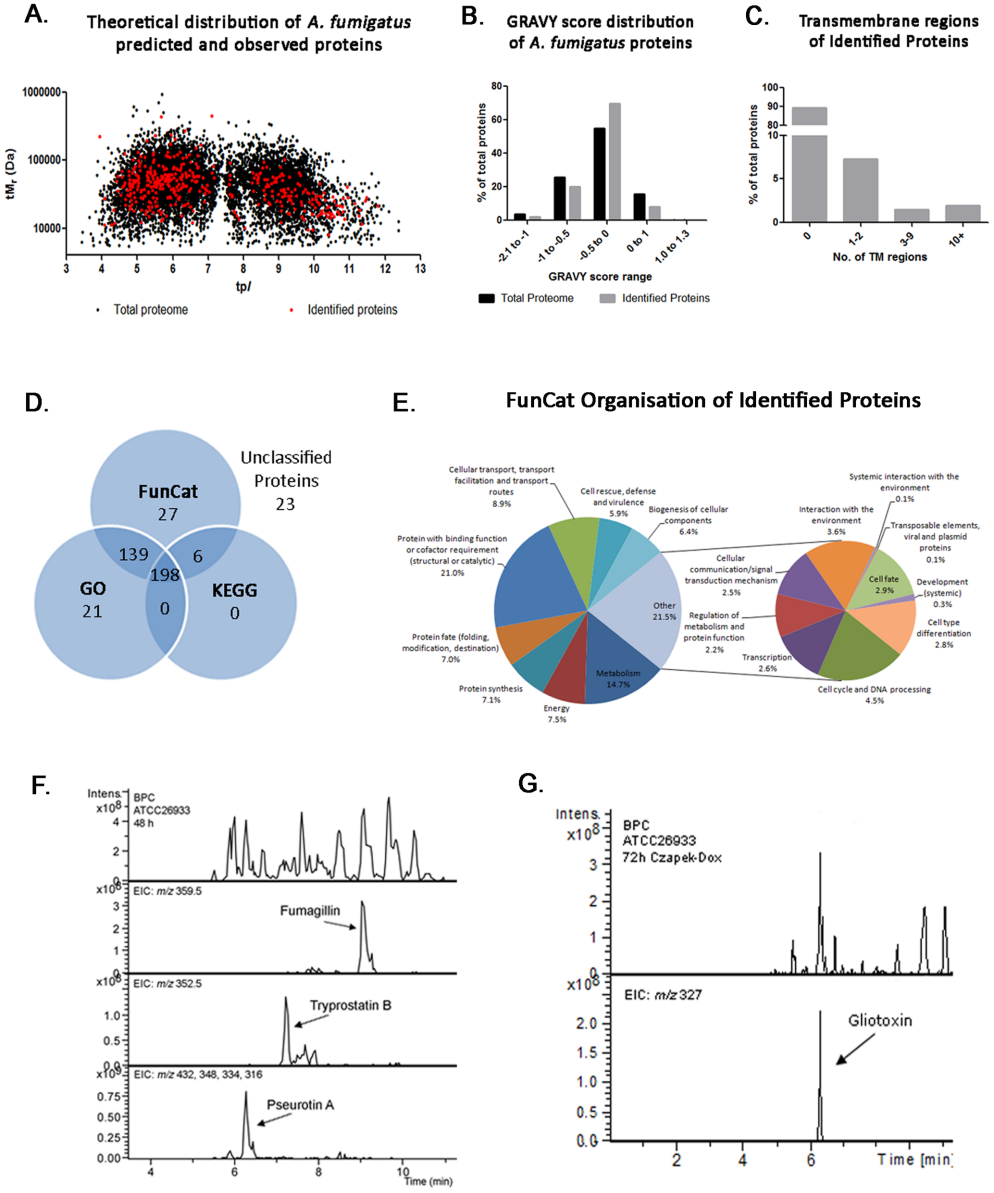
Overview of *A. fumigatus* shotgun proteomic data. (a) Proteome map showing distribution of *A. fumigatus* proteins based on theoretical M_r_ and p*I* where proteins identified by shotgun mass spectrometry (*n* = 414; red) are shown overlaid on the total *A. fumigatus* proteome (black). tM_r_, theoretical molecular mass, axis drawn on logarithmic scale; tp*I*, theoretical isoelectric point, axis drawn on linear scale. (b, c) Distribution of proteins identified by shotgun mass spectrometry (MS) according to their relative hydrophobicity and the number of putative transmembrane regions per protein. The number of putative transmembrane regions on each protein identified by shotgun MS is shown. (d) Distribution of functional annotations of *A. fumigatus* proteins identified using shotgun proteomics strategy. GO, KEGG and FunCat classification schemes were used for functional annotation utilizing the FungiFun application. A number of proteins (*n* = 23) were identified that possessed no functional classification using this system. (e) The functional categorization of the proteins identified here, based on the FunCat annotation scheme, are shown. *Note:* GRAVY, grand average of hydropathy; TM, transmembrane; MS, mass spectrometry. (f) LC-MS detection of SM in *A. fumigatus* organic extracts from AMM cultures and (g) Czapek-Dox cultures (BPC: Base Peak Chromatogram; EIC: Extracted Ion Chromatogram).

Utilising a direct shotgun proteomics approach, a total of 1826 unique peptides were identified in *A. fumigatus* mycelial lysates, corresponding to 414 distinct *A. fumigatus* proteins from 405 protein groups (Table S1). A false discovery rate (FDR) of 1.97% was determined for the distinct peptides identified in this study, using the validation criteria outlined (Table S1). These proteins (*n* = 414) spanned a theoretical p*I* range of 3.9 to 11.8 and a M_r_ range of 7.8 to 444 kDa (Figure 1; Table S1). All peptides identified contributed to a sequence coverage range of between 1 and 64% of the respective proteins, with Spectrum Mill scores ranging between 17 and 1327.

The GRAVY index for identified proteins ranged from −1.632 to 0.483, with positive scores indicating hydrophobicity (Figure 1). A number of hydrophobic proteins were identified (*n* = 33; 7.97% of total identified proteins), based on positive GRAVY scores. Additionally, GRAVY scores were computed for the entire predicted proteome of *A. fumigatus* and it was observed that 15.6% of the total proteome possess positive GRAVY scores (Figure 1). The majority of proteins identified by shotgun mass spectrometry (70.05%) were slightly hydrophilic, with GRAVY scores ranging from −0.5 to 0. This is in accordance with the total predicted proteome of *A. fumigatus*, where 55.1% of all predicted proteins fall within this range. Proteins with transmembrane helices (*n* = 44; 10.62% of total identified proteins) were detected. Several proteins were detected with 10 or more putative TM regions, including a plasma membrane H^+^-ATPase (AFUA_3G07640), an amino acid permease (Gap1) (AFUA_7G04290) and two ABC transporters (AFUA_1G14330 and AFUA_5G06070). One protein, a small oligopeptide transporter (OPT family) (AFUA_2G15240) was detected with 14 putative transmembrane regions and a GRAVY score of 0.276. Annotations were available for 89.37%, 86.47% and 49.27% of identified proteins using the FunCat, GO and KEGG schemes, respectively (Figure 1). Based on the FunCat classification, functional categories that were significantly over-represented were protein synthesis (*n* = 86, *p* = 4.68×10^−23^), energy (*n* = 91, *p* = 4.22×10^−17^), protein with binding function or cofactor requirement (*n* = 254, *p* = 4.43×10^−14^) and transcription (*n* = 32, *p* = 2.79×10^−9^). Proteins (*n* = 23; 5.5%) were identified which have no functional classifications using the aforementioned methods.

### Identification of *A. fumigatus* secondary metabolite cluster expression at protein level

Proteins identified by shotgun mass spectrometry were mapped based on their relative loci on each of the eight *A. fumigatus* chromosomes, using their gene locus identifiers (Figure S1). A number of proteins (*n* = 15) that comprise a secondary metabolite supercluster, involved in the production of pseurotin A, fumitremorgins and fumagillin [38]–[42], were identified (Table 1). In addition, proteins were identified from the gliotoxin biosynthetic cluster on chromosome 6 [43], including GliT, the gliotoxin oxidoreductase responsible for self-protection against gliotoxin [28], and two clusters responsible for the production of unknown metabolites on chromosomes 3 and 4 respectively (Table 1). A phosphoglycerate kinase PgkA protein (AFUA_1G10350) was also identified, which is predicted to be part of the *Afpes1* NRPS cluster on chromosome 1 [1]. The identification of these proteins is indicative of the respective cluster activity under the growth conditions used. To confirm whether this detection of secondary metabolism-associated proteins correlated with the production of the respective molecules, LC-MS analysis was carried out on culture supernatants. This analysis revealed the presence of fumagillin, tryprostatin B and pseurotin A, along with an array of other, as yet unidentified, molecules (Figure 1F). These secondary metabolites are all products of the ‘supercluster’ on Chromosone 8 [44]–[46], demonstrating correlation between the proteomic and metabolomic profiles. Interestingly, expression of the clusters identified here is partially or completely regulated by the transcription regulator LaeA [38]. Two additional proteins, encoded by AFUA_3G03280 and AFUA_3G03330, were also detected, from a cluster with predicted involvement in the production of a siderophore and a toxin [38] (Table 1). Following on from this observation of SM cluster protein detection, mycelia from cultures grown in Czapek-Dox media for 72 h were also analysed. Shotgun proteomic analysis revealed the presence of four proteins from the gliotoxin biosynthetic cluster under the conditions used. The identification of peptides from GliN, GliF, GliH and GliT (Table S2), correlated with the presence of gliotoxin in the culture supernatants of these cultures (Figure 1G).

**Table 1:** *A. fumigatus* proteins, involved in secondary metabolism, and identified by shotgun mass spectrometry.

Cluster No^a^	CADRE ID. (AFUA_)	Protein name	Chromosome No	LaeA regulation^a^	Product(s)
1	**1G10350**	Phosphoglycerate kinase PgkA (EC 2.7.2.3)	1	Yes	Fumigaclavine C
7	**3G03280**	FAD binding monooxygenase	3	No	Putatively two products: a siderophore and a toxin
7	**3G03330**	Mitochondrial enoyl reductase	3	No	
10	**3G14680**	Lysophospholipase 3 (EC 3.1.1.5) (Phospholipase B 3)	3	Partial	Unknown
13	**4G14380**	Glutathione S-transferase, putative	4	Partial	Unknown
18	**6G09740**	GliT (Thioredoxin reductase GliT) (EC 1.-.-.-)	6	Yes	Gliotoxin
22	**8G00230**	Phytanoyl-CoA dioxygenase family protein	8	Yes	‘Supercluster’ producing Fumitremorgin B, Pseurotin A and Fumagillin
22	**8G00370**	Polyketide synthase, putative	8	Yes	
22	**8G00380**	DltD N-terminal domain protein	8	Yes	
22	**8G00390**	O-methyltransferase, putative	8	Yes	
22	**8G00400**	Unknown function protein	8	Yes	
22	**8G00430**	Unknown function protein	8	Yes	
22	**8G00440**	Steroid monooxygenase, putative (EC 1.-.-.-)	8	Yes	
22	**8G00480**	Phytanoyl-CoA dioxygenase family protein	8	Yes	
22	**8G00500**	Acetate-CoA ligase, putative (EC 6.2.1.1)	8	Yes	
22	**8G00510**	Cytochrome P450 oxidoreductase OrdA-like, putative	8	Yes	‘Supercluster’ producing Fumitremorgin B, Pseurotin A and Fumagillin
22	**8G00530**	Alpha/beta hydrolase, putative	8	Yes	
22	**8G00540**	Hybrid PKS-NRPS enzyme, putative	8	Yes	
22	**8G00550**	Methyltransferase SirN-like, putative	8	Yes	
22	**8G00580**	Glutathione S-transferase, putative	8	Yes	

CADRE ID., *A. fumigatus* gene annotation nomenclature according to [1] and [71].

a Cluster numbers and LaeA regulation as denoted in [38].

### Comparative 2-D analysis of *A. fumigatus* ATCC26933 following exposure to a combination of gliotoxin and H_2_O_2_

**Figure 2:**
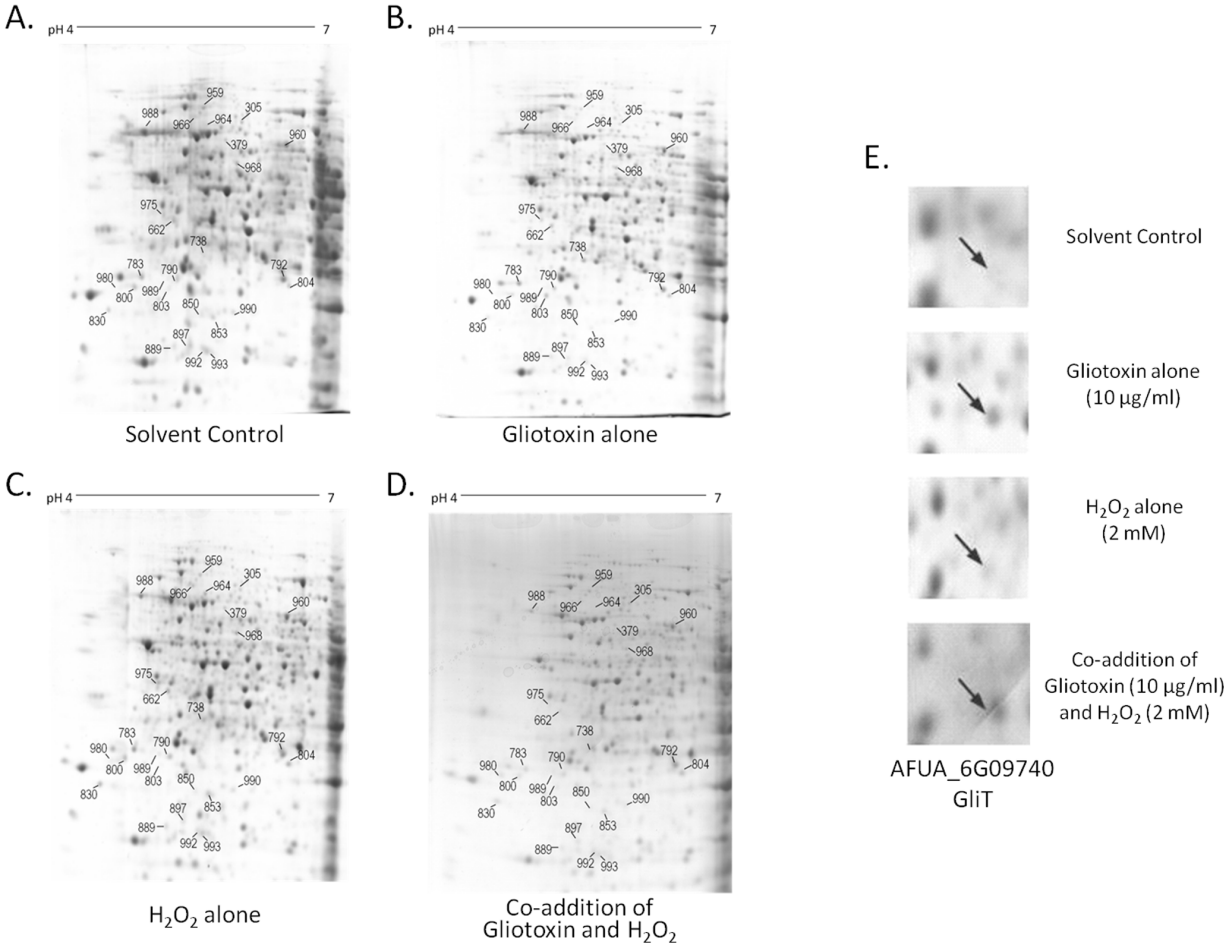
2-D analysis reveals differential proteomic response *of A. fumigatus* to a combination of gliotoxin and H_2_O_2_. 2-D proteome maps of *A. fumigatus* ATCC26933 (a) solvent control, (b) following exposure to gliotoxin (10 µg/ml) for 4 h, (c) following exposure to 2 mM H_2_O_2_ for 4 h, (d) following exposure to a combination of gliotoxin (10 µg/ml) and H_2_O_2_ (2 mM) for 4 h. The proteins were first separated on pH 4–7 strips followed by SDS-PAGE. Proteins found to be significantly differentially expressed (*p*<0.05), after analysis using Progenesis SameSpot software, are numbered. (e) Increased expression of the gliotoxin oxidoreductase GliT in response to gliotoxin but not H_2_O_2_. GliT expression was increased following exposure to exogenous gliotoxin alone (5.1 fold) and in combination with H_2_O_2_ (4.8 fold), relative to the solvent control. No significant difference in expression of GliT was detected upon exposure of *A. fumigatus* to H_2_O_2_ alone, relative to the control (*p*>0.05), indicating GliT expression is mediated by gliotoxin only.

Proteins (*n* = 13) were found to be significantly differentially abundant (*p*<0.05) when *A. fumigatus* was co-exposed to gliotoxin/H_2_O_2_ compared to H_2_O_2_ alone (Figure 2; Table 2). These comprised six proteins with an abundance increase, and seven proteins with a decrease, of at least 1.5 fold. Furthermore, comparative analyses were carried out on all comparitor sets, to identify alterations in protein abundance between individual treatments (i.e., gliotoxin alone, H_2_O_2_ alone, co-addition and solvent control). Redundancy was noted, with some proteins included in multiple comparison sets, resulting in the net differential expression of 27 unique proteins (Figure 2). These proteins were excised and subjected to in-gel trypsin digestion, followed by LC-MS/MS analysis for protein identification.

**Table 2:** Proteins (n = 13) exhibiting significant differential abundance1 in A. fumigatus ATCC26933 following the co-addition of gliotoxin and H2O2, relative to H2O2 alone.

Protein Name	Co-addition v Control (iii)	Co-addition v Gliotoxin (iv)	Co-addition v H_2_O_2_ (v)	H_2_O_2_ v Control	CADRE ID. (AFUA_)	Spot No.
**Increased Abundance Proteins in Co-addition v H_2_O_2_**						
Thioredoxin reductase GliT	↑ 4.8	-	↑ **3.5**	-	6G09740	738
Unknown function protein	↑ 4.4	-	↑ **4.9**	-	2G11120	803
Proliferating cell nuclear antigen (PCNA)	↑ 5.9	↑ 7.4	↑ **2.4**	-	1G04900	980
Ran-specific GTPase-activating protein 1	-	-	↑ **1.8**	-	5G12180	850
NADH-quinone oxidoreductase (23 kDa subunit)	-	-	↑ **1.9**	-	1G06610	897
HAD family hydrolase	↑ 1.8	↑ 2.1	↑ **1.5**	-	5G08270	989
**Decreased Abundance Proteins in Co-addition v H_2_O_2_**						
Unknown function protein	-	-	↓ **2.9**	↑ 2.3	6G03460	964
Molecular chaperone and allergen Mod-E/Hsp90/Hsp1	↓ 1.5	-	↓ **2.7**	↑ 2.0	5G04170	966
Oxidative stress protein Svf1	-	-	↓ **1.6**	-	5G11820	975
Glutamine amidotransferase: cyclase	↓ 1.9	-	↓ **1.7**	-	2G06230	968
Glycyl-tRNA synthetase	↓ 1.9	-	↓ **1.5**	-	5G05920	305
Methylenetetrahydrofolate reductase	↓ 1.9	-	↓ **1.6**	-	2G11300	379
Class V chitinase	-	-	↓ **1.8**	-	3G11280	662

Data extracted from Tables S3 and S4 and re-charted for clarity. Proteins detected with a significant change in abundance in H_2_O_2_ compared to the control are also reported.

1 *p*<0.05; Fold increase (↑) or decrease (↓) of protein in the co-additive condition, relative to the solvent control, gliotoxin alone or H_2_O_2_ alone. Co-addition: incubation with both gliotoxin and H_2_O_2_. CADRE ID., *A. fumigatus* gene annotation nomenclature according to [1] and [71]; Spot No, according to Figure 2. Numbers in bold indicate fold change of proteins (*n* = 13) differentially regulated in the co-addition, relative to H_2_O_2_ alone.

### Identification of differentially abundant proteins by LC-MS/MS

LC-MS/MS analysis was used to identify the 27 proteins which were differentially abundant following challenges with gliotoxin and H_2_O_2_, individually or in combination (Tables S3 and S4, Table 2). Protein abundance was assessed for all conditions relative to the solvent control (Tables S3 and S4). The abundance of proteins after gliotoxin/H_2_O_2_ co-exposure was also assessed relative to the individual treatments of gliotoxin alone or H_2_O_2_ alone (Table 2). Proteins (*n* = 13) were significantly altered in abundance following co-addition relative to H_2_O_2_ alone (*p*<0.05) (Table 2, Figure 2). Proteins exhibiting increased abundance in the co-addition included those with oxidation-reduction activity. GliT, the gliotoxin oxidoreductase [28], [47], was more abundant in the co-addition condition relative to H_2_O_2_ alone (3.5 fold) but was unaltered by H_2_O_2_ addition alone (Figure 2, Table 2 and Table S4). An increase in abundance of the Ran-specific GTPase and the proliferating cell nuclear antigen (PCNA), involved in cell-cycle regulation and DNA-repair [48], [49], respectively, was also observed in the presence of gliotoxin/H_2_O_2_ together. The HAD family hydrolase, also exhibited increased abundance when both gliotoxin and H_2_O_2_ were present, relative to any of the control conditions. Proteins involved in amino acid and nucleic acid metabolism [50], [51], glutamine amidotransferase: cyclase and methylenetetrahydrofolate reductase, also showed differential abundance. The class V chitinase, associated with cell autolysis [52], was significantly less abundant (*p* = 5.2×10^−5^) upon co-addition relative to H_2_O_2_ alone. A decrease in abundance of proteins associated with response to stress was observed following gliotoxin/H_2_O_2_ co-exposure, relative to H_2_O_2_ alone. Hsp90 and the oxidative stress protein Svf1 were of lower abundance in the co-addition (2.7 and 1.6 fold, respectively), reflective of the relief of H_2_O_2_-induced stress (Table 2, Figure 2). Additionally two unknown function proteins were detected, which underwent a 4.9 fold increase (AFUA_2G11120) and a 2.9 fold decrease (AFUA_6G03460) in abundance in the co-addition, relative to H_2_O_2_ alone. This latter observation underpins the necessity to undertake both shotgun and 2-D based approaches to identify novel proteins.

### Gliotoxin inhibits H_2_O_2_-induced ROS formation in *A. fumigatus*

**Figure 3:**
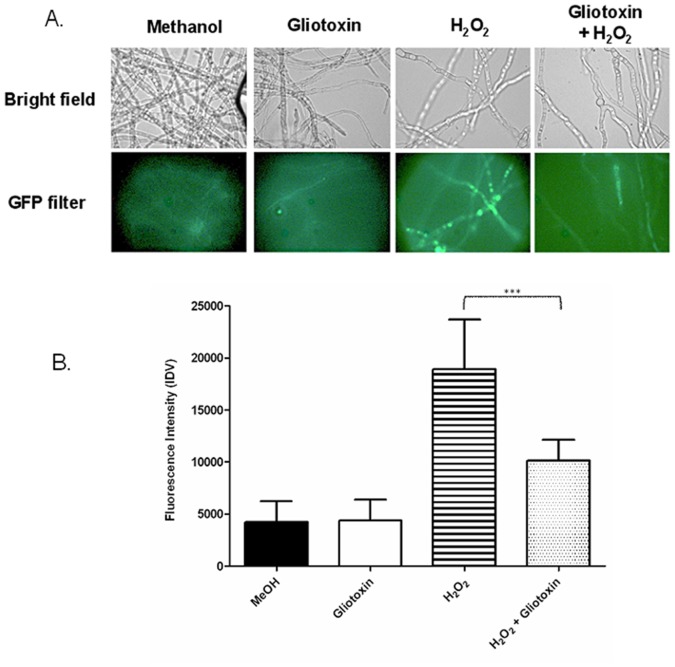
Gliotoxin attenuates H_2_O_2_-induced ROS formation. (a) Neither methanol (solvent control) or gliotoxin induce significant ROS formation in *A. fumigatus*, however H_2_O_2_ exposure leads to clear formation of ROS. Co-addition of gliotoxin dissipates ROS as judged by reduced fluorescence. (b) Gliotoxin significantly reduces H_2_O_2_-induced ROS levels during co-incubation with H_2_O_2_ (*p*>0.0001).

Previous work has demonstrated that GliT is essential to protect *A. fumigatus* against exogenous gliotoxin [28] but contra-intuitively, that gliotoxin reverses H_2_O_2_-mediated growth inhibition of *A. fumigatus* ATCC26933 and Δ*gliK* ^26933^ [30], yet, no mechanistic explanation was forthcoming. Results from the comparative proteomics analysis indicated that gliotoxin may effect an alleviation of H_2_O_2_ induced-oxidative stress. Data in Figure 3 show ROS formation consequent to H_2_O_2_ exposure in *A. fumigatus*. However, co-addition of gliotoxin results in a significant reduction (*p*>0.0001) in the production of reactive metabolites, as judged by decreased 2′,7′-dichlorofluorescein fluorescence. This suggests that gliotoxin acts as an anti-oxidant and functions to impede H_2_O_2_-mediated growth inhibition.

## Discussion

A comprehensive mycelial proteome reference map, produced by Vödisch *et al.* [53], identified proteins with a GRAVY score up to 0.158 and fourteen proteins with 1–2 putative TM regions, which computes to 4.2% of the identified proteins possessing TM regions. Here, by comparison, 44 proteins possessing predicted TM domains were identified from *A. fumigatus* mycelia, corresponding to 10.62% of the proteins identified using shotgun mass spectrometry alone (Table S1). This represents a substantial increase (2.5 fold) in the identification of proteins with TM regions, compared to previous 2-D based studies with similar targets [6]. Using the shotgun proteomics approach, 33 hydrophobic proteins, corresponding to 7.97% of total identified proteins, were detected, including a protein transport protein SEC61 alpha subunit (AFUA_5G08130) with a GRAVY score of 0.4828, compared to 3.4% hydrophobic protein content by previous 2-D coupled MS analysis [53]. The standard molecular mass resolution of *A. fumigatus* mycelial proteins, using 2-D, ranges from 10 to 142 kDa [3], [4], [6]. The constraint of high molecular mass did not apply to the shotgun proteomic approach used in this study, with the identification of 12 proteins possessing a molecular mass greater than 142 kDa. The largest protein detected was encoded by AFUA_5G02570, with predicted histone acetyltransferase activity and a theoretical mass of 444 kDa. PesO [54], a hybrid polyketide synthase/ non-ribosomal peptide synthetase (PKS/NRPS) (AFUA_8G00540), with a theoretical molecular mass of 434 kDa was also identified. Here, 73 unique peptides were identified from this protein, contributing to sequence coverage of 28%. PesO is involved in the production of pseurotin A [40] and its identification provides evidence of expression of this secondary metabolite cluster. A 267 kDa polyketide synthase (AFUA_8G00370) was also identified by 10 unique peptides, contributing to 10% sequence coverage. These findings represent some of the largest *A. fumigatus* proteins to be identified by mass spectrometry to date. Cagas *et al.* [9] utilised iTRAQ in order to profile the early development proteome of *A. fumigatus*. This gel-free method of large scale proteomic identification extended the molecular mass limits of detection to 9 to 255 kDa, thereby confirming the value of alternative methods for proteomic investigation. Supplementary information (Text S1) provides further discussion of shotgun MS data.

Verification of proteins encoded by genes within secondary metabolite clusters was achieved in this study using shotgun mass spectrometry. Proteins identified from AMM cultures are putatively encoded by six clusters involved in the production of up to nine secondary metabolites (SM) [38]. Products of these SM clusters include up to 3 unknown metabolites, fumigaclavine C [55], fumitremorgins [39], [44], [45], pseurotin A [40], fumagillin [41], [46], gliotoxin [43] and a putative siderophore [38]. Perrin *et al.* [38] annotated a ‘supercluster’ on Chromosome 8 (AFUA_8G00100-AFUA_8G00720) that is involved in the production of fumitremorgins, pseurotin A and fumagillin [39], [40], [46]. Fifteen proteins identified by shotgun mass spectrometry are annotated members of this ‘supercluster’, with one identified protein involved in the production of fumitremorgins and tryprostatins, four proteins involved in the pseurotin A biosynthetic portion of the cluster and the remaining nine proteins associated with fumagillin biosynthesis. Metabolomic investigation confirmed the presence of fumagillin, tryprostatin B and pseurotin A in culture supernatants, confirming the activity of these clusters, as indicated by shotgun proteomics. Phytanoyl-CoA dioxygenase family protein (FtmF) (AFUA_8G00230) was identified by 3 unique peptides and a sequence coverage of 14%. FtmF, a non-heme Fe(II) and α-ketoglutarate-dependent dioxygenase, catalyses the conversion of fumitremorgin B to verruculogen via endoperoxide bond formation [56]. This enzyme is also capable of converting fumitremorgin B to 12α,13α-dihydroxyfumitremorgin C and 13-oxo-verruculogen, by deprenylation and oxidation mechanisms respectively [57]. Verruculogen, like fumitremorgin B, is a tremorgenic mycotoxin and has been shown to produce deleterious effects on respiratory epithelial cells [58]. A second protein (AFUA_8G00280) was also identified from the border region of this cluster, with putative oxidoreductase activity, and along with the detection of FtmF, suggests the production of fumitremorgins or tryprostatins by *A. fumigatus* under the conditions of culture. Pseurotin A production is also encoded by the ‘supercluster’ on Chromosome 8 [40], and four enzymes, that form part of the pseurotin biosynthetic cluster, were detected here; an alpha/beta hydrolase (AFUA_8G00530), a hybrid PKS-NRPS enzyme PesO (AFUA_8G00540), a methyltransferase SirN-like (AFUA_8G00550) and a putative glutathione S-transferase (AFUA_8G00580) [4]. This cluster has demonstrated increased expression at both the transcript and protein level under hypoxic conditions [21]. Furthermore, up-regulation of the methyltransferase and PesO transcripts were also shown in the mouse lung during infection by *A. fumigatus* [21]. Identification of nine proteins from the portion of the supercluster associated with fumagillin biosynthesis, represents significant coverage of this fifteen member cluster by shotgun mass spectrometry. Fumagillin is a meroterpenoid, with demonstrated anti-angiogenic activity through interaction with methionine aminopeptidase II (MetAP2) [59]. Fumagillin has also been associated with disruption of NADPH oxidase function could represent a putative virulence factor [60]. Indeed, cognate transcript expression of six of the proteins identified from this cluster was up-regulated in *A. fumigatus* Af293 during the initiation of murine infection [61]. Identification of a number of proteins from both the pseurotin A and fumagillin clusters is in-line with the recent identification of a transcription factor, FapR, which co-regulates expression of genes in these two clusters [46]. Further investigation of a second minimal media culture condition (Czapek-Dox, 72 h) revealed a similar observation. Proteins (*n* = 4) from the gliotoxin biosynthetic cluster were identified (Table S2), in line with the detection of gliotoxin in culture supernatants (Figure 1G). Enlisting a shotgun proteomic approach provides a non-targeted method to detect the expression of proteins involved in secondary metabolism, in any given growth condition, and could prove useful as a tool for the identification of novel metabolites.

Proteomics also revealed changes in protein abundance associated with SM (gliotoxin)-mediated relief of H_2_O_2_-induced stress. Proteins (*n* = 13) were differentially abundant following exposure to a combination of H_2_O_2_ (2 mM) and gliotoxin (10 µg/ml), relative to H_2_O_2_ alone (2 mM), which facilitates dissection of the mechanisms involved in gliotoxin-mediated relief of H_2_O_2_-induced stress (Table 2). Increased abundance of two proteins, in response to gliotoxin and H_2_O_2_ in combination, relative to H_2_O_2_ alone, with predicted or demonstrated oxidoreductase activity included the gliotoxin oxidoreductase GliT and the NADH-quinone oxidoreductase (23 kDa subunit), with 3.5 and 1.9 fold increase in abundance, respectively. In addition to a key role in the gliotoxin biosynthetic process, GliT also mediates self-protection against the harmful effects of gliotoxin [28], [47]. Increased expression of GliT was detected following exposure to gliotoxin alone (5.1 fold), as previously noted [20], [28] and combined with H_2_O_2_ (4.8 fold) relative to the solvent control (Figure 2). There was no significant alteration to abundance of GliT in response to H_2_O_2_ alone (*p* = 0.297) and this demonstrates that GliT abundance is not regulated by H_2_O_2_ and increased levels in the co-addition condition is solely a result of gliotoxin presence. Choi *et al.* [62] noted that gliotoxin catalysed H_2_O_2_ reduction, mediated by the mammalian thioredoxin redox system and proposed that gliotoxin replaces 2-cys peroxiredoxin as an electron acceptor, in the reduction of H_2_O_2_ to H_2_O in mammalian cells.

The proliferating cell nuclear antigen (PCNA) exhibited a 2.4 fold increase in abundance following incubation with a combination of gliotoxin and H_2_O_2_, relative to H_2_O_2_ alone. Moreover, abundance of this protein was also further increased in the co-addition condition, relative to gliotoxin alone (7.4 fold) and the solvent control (5.9 fold; Table 2). Thus, PCNA abundance is induced by H_2_O_2_ (approximately 3.5 fold) and not by exposure to gliotoxin alone. Clearly, a combination of H_2_O_2_ with gliotoxin leads to further induction of this protein. PCNA is involved in the process of DNA-repair following H_2_O_2_-mediated damage [48] and acts as an anchor to the DNA template for binding partners [63]. The increase in PCNA abundance observed in response to H_2_O_2_, alone or coupled with gliotoxin, may therefore be indicative of H_2_O_2_-induced DNA damage in these conditions. Furthermore, additional induction of PCNA abundance in the co-addition condition relative to H_2_O_2_, alone, may account for the recovery of growth, due to enhanced DNA repair capacity.

Proteins involved in the response to cellular stress underwent decreased abundance in the co-addition condition, relative to H_2_O_2_ alone. A decrease in abundance of Hsp90 (2.7 fold) and the oxidative stress protein Svf1 (1.6 fold) was noted. Hsp90 displayed increased abundance in the presence of H_2_O_2_ alone, relative to the solvent control (2.0 fold), with this response reversing upon co-incubation with gliotoxin. In accordance with these observations at the proteomic level, the transcript of Hsp90 was also reported to be up-regulated in *A. fumigatus* in response to exogenous H_2_O_2_ [64]. Hsp90 is a stress-induced protein involved in the refolding of denatured proteins and signal transduction [64], [65]. The decrease in abundance of Hsp90 is indicative of the decrease in oxidative stress, correlating with the relief of growth inhibition observed. The decreased abundance of Svf1, with a nuclear localisation and a role in the response to oxidative stress [7], [66], is also diagnostic for the attenuation of oxidative stress in the co-addition condition relative to H_2_O_2_ alone. Indeed, a significant reduction (*p*<0.0001) in ROS levels was detected following co-application of gliotoxin and H_2_O_2_, relative to H_2_O_2_ alone (Figure 3), thus providing biochemical verification of the proteomics data. A decrease in abundance (1.8 fold) of the class V chitinase in the presence of a combination of gliotoxin and H_2_O_2_ was observed, relative H_2_O_2_ alone. This protein belongs to subgroup A of fungal/bacterial chitinases which are associated with fungal growth and autolysis [67], [68]. The orthologous *A. nidulans* protein, ChiB, has demonstrated involvement in the autolysis of fungal mycelia in response to stress [52]. A higher abundance of this protein, in the presence of H_2_O_2_ alone, may indicate the occurrence of mycelial autolysis, which could have been stimulated by the presence of oxidative stress. This autolysis could also account for the growth-inhibited phenotype observed in the presence of H_2_O_2_ alone [30].

**Figure 4:**
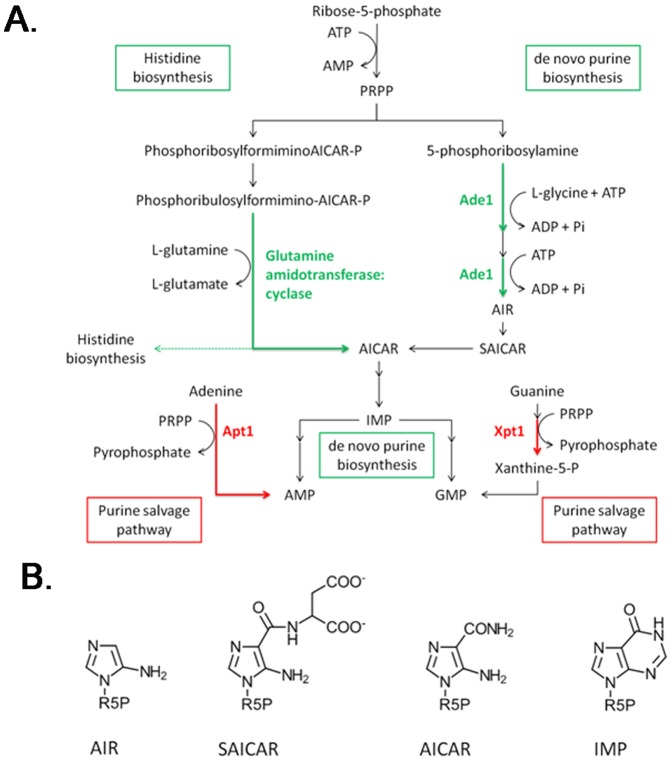
Overview of the regulation of the purine metabolic pathway by gliotoxin and H_2_O_2_, either alone or in combination. (a) Purine-related enzymes and pathways undergoing an increase in expression, relative to the solvent control, are indicated in red and decreased expression is indicated in green. Metabolites are indicated in black. Ade1, bifunctional purine biosynthetic protein; Xpt1, xanthine-guanine phosphoribosyltransferase; Apt1, adenine phosphoribosyltransferase. Enzymes of the histidine and *de novo* purine biosythesis converging pathways, glutamine amidotransferase: cyclase and Ade1, are down-regulated in response to gliotoxin. Expression of enzymes involved in the purine salvage pathways, Xpt1 and Apt1, is up-regulated in the presence of H_2_O_2_ and gliotoxin, repsectively, relative to a solvent control [20], [23]. Figure adapted from pathway.yeastgenome.org. (b) Structures of intermediate molecules in the purine and histidine biosynthesis pathway; 5-aminoimidazole ribonucleotide (AIR), *N*-succinyl-5-aminoimidazole-4-carboxamide ribonucleotide (SAICAR), 5-aminoimidazole-4-carboxamide ribonucleotide (AICAR) and inosine monophosphate (IMP). R5P  =  ribulose-5-phosphate.

Proteins involved in amino acid and nucleotide metabolism also decreased in abundance in the presence of gliotoxin and H_2_O_2_ combined, relative to H_2_O_2_ alone. Glutamine amidotransferase: cyclase and methylenetetrahydrofolate reductase underwent a 1.7 and 1.6 fold decrease in abundance, respectively, relative to H_2_O_2_ alone. Additionally, abundance of both of these proteins was decreased 1.9 fold in the co-addition condition, relative to the solvent control. Considering these observations, H_2_O_2_ does not appear to be involved in the controlling the levels of these proteins. Instead, gliotoxin, either independently or in combination with H_2_O_2_, is responsible for triggering the decrease in abundance of these proteins. Glutamine amidotransferase: cyclase catalyses two steps in the biosynthesis of histidine, producing both a histidine precursor and 5-aminoimidazole-4-carboxamide ribonucleotide (AICAR), an intermediate of the purine biosynthetic process, thus linking these pathways [50]. Interestingly, the bifunctional purine biosynthetic protein, Ade1, was reduced in abundance in the presence of gliotoxin relative to the solvent control (1.8 fold) (Figure 2). Conversely, xanthine-guanine phosphoribosyl transferase Xpt1, was more abundant in the presence of gliotoxin and H_2_O_2_ combined, relative to the solvent control (1.8 fold) and gliotoxin alone (1.6 fold), indicating that levels of this protein are influenced by H_2_O_2_. Indeed, Lessing *et al.* [23] observed an increase in abundance of Xpt1 following exposure to H_2_O_2_ for 45 min. Xpt1 is involved in the purine nucleotide salvage pathway, whereby XMP and GMP are formed from precursors, xanthine and guanine, respectively [69] (Figure 4; Table 3). Additionally, the increased abundance of another component of the purine salvage pathway, adenine phosphoribosyltransferase, has been noted in response to exogenous gliotoxin [20]. These observations reveal a diminution of *de novo* purine biosynthesis in the presence of gliotoxin and that the alternative salvage pathway is utilised in its place (Figure 4; Table 3). Together, these observations underline the influence of gliotoxin and H_2_O_2_, either alone or in combination, on nucleotide biosynthesis in *A. fumigatus*.

**Table 3:** Summary of abundance changes of proteins involved in purine biosynthesis pathways.

Protein Name	Gliotoxin v Control (i)	H_2_O_2_ v Control (ii)	Co-addition v Control (iii)	Co-addition v Gliotoxin (iv)	Co-addition v H_2_O_2_ (v)	CADRE ID. (AFUA_)	Spot No.
**Proteins involved in purine salvage pathway**							
Adenine phosphoribosyl transferase Apt1	↑ 20.5*					7G02310	N/A
Xanthine-guanine phosphoribosyl transferase Xpt1		↑ 3.4*	↑ 1.8	↑ 1.6		4G04550	992
**Proteins involved in de-novo purine biosynthesis**							
Glutamine amidotransferase: cyclase			↓ 1.9		↓ 1.7	2G06230	968
Bifunctional purine biosynthesis protein Ade1	↓ 1.8					6G04730	959

Fold increase (↑) or decrease (↓) of protein, relative to the respective control. CADRE ID., *A. fumigatus* gene annotation nomenclature according to [1] and [71]; Spot No, according to Figure 2.

*Change in protein abundance was reported previously [20], [23].

Furthermore, while no definitive functions have been demonstrated for the unknown function proteins encoded by AFUA_6G03460 and AFUA_2G11120, computational analysis has assigned the function of D-alanine-D-alanine ligase and methyltransferase to these proteins, respectively [32]. An orthologue of this methyltransferase (MT-II) was found to be up-regulated in *A. niger* in response to reductive stress from DTT [70], which may resemble the stress induced by gliotoxin and presents an interesting target for future investigations.

## Conclusions

In summary, shotgun proteomics has revealed expression of multiple proteins involved in secondary metabolite biosynthesis coincident with detection of the cognate metabolites, and provides strong evidence for the activation of multiple clusters under the control of the transcriptional regulator LaeA, in the conditions tested. Our findings also demonstrate how proteomics can inform how the SM, gliotoxin, effects attenuation of H_2_O_2_-mediated oxidative stress.

## Supporting Information

Figure S1 **Distribution of proteins identified using shotgun mass spectrometry (** ***n*** **= 414) based on gene locus (blue lines). Identification of proteins (** ***n*** **= 15) from a supercluster on chromosome 8, involved in the production of fumitremorgin B, pseurotin A and fumagillin (red circle).**
(DOC)

Table S1 **Proteins identified by shotgun MS analysis.**
(XLS)

Table S2 **Peptides detected from gliotoxin cluster proteins following growth in Czapek-Dox media for 72 h at 37°C.**
(XLS)

Table S3 **Proteins undergoing significant differential abundance^1^ in** ***A. fumigatus*** **ATCC26933 following exposure to gliotoxin and H_2_O_2_, separately or combined, relative to the solvent control.** Protein identification was achieved by 2D-PAGE and LC-MS/MS.
(DOC)

Table S4 **Proteins undergoing significant differential abundance^1^ in** ***A. fumigatus*** **ATCC26933 following exposure to a combination of gliotoxin and H_2_O_2_ (co-addition), relative to the control, gliotoxin alone or H_2_O_2_ alone.** Protein identification was achieved by 2D-PAGE and LC-MS/MS.
(DOC)

Text S1 **Supplementary Discussion.**
(DOC)
