# Supplementary material for: A Proteomic Approach to Investigating Gene Cluster Expression and Secondary Metabolite Functionality in Aspergillus fumigatus
Source: PLoS One. 2014 Sep 8;9(9):e106942. doi: 10.1371/journal.pone.0106942 (PMC4157829; doi:10.1371/journal.pone.0106942)
Supplement: Figure S1 — Distribution of proteins identified using shotgun mass spectrometry ( n = 414) based on gene locus (blue lines). Identification of proteins ( n = 15) from a supercluster on chromosome 8, involved in the production of fumitremorgin B, pseurotin A and fumagillin (red circle). (DOC) [file pone.0106942.s001.doc]

**Supplementary Information**

**A Proteomic Approach to Investigating Gene Cluster Expression and Secondary Metabolite Functionality in *Aspergillus fumigatus*.**

Rebecca A. Owens, Stephen Hammel, Kevin J. Sheridan, Gary W. Jones and Sean Doyle*.

Department of Biology, National University of Ireland Maynooth, Maynooth, Co. Kildare, Ireland.

***Corresponding author**

Professor Sean Doyle,

Department of Biology,

National University of Ireland Maynooth,

Maynooth, Co. Kildare, Ireland.

Tel: +353-1-7083858; Fax: +353-1-7083845; E-mail: sean.doyle@nuim.ie

Web: http://biology.nuim.ie

**Keywords**

Fungal proteomics, gliotoxin, redox stress, NRPS, mycotoxin, LC-MS

**Figure S1:** Distribution of proteins identified using shotgun mass spectrometry (*n* = 414) based on gene locus (blue lines). Identification of proteins (*n* = 15) from a supercluster on chromosome 8, involved in the production of fumitremorgin B, pseurotin A and fumagillin (red circle).
