# Supplementary material for: A Proteomic Approach to Investigating Gene Cluster Expression and Secondary Metabolite Functionality in Aspergillus fumigatus
Source: PLoS One. 2014 Sep 8;9(9):e106942. doi: 10.1371/journal.pone.0106942 (PMC4157829; doi:10.1371/journal.pone.0106942)
Supplement: Table S3 — Proteins undergoing significant differential abundance1 in A. fumigatus ATCC26933 following exposure to gliotoxin and H2O2, separately or combined, relative to the solvent control. Protein identification was achieved by 2D-PAGE and LC-MS/MS. (DOC) [file pone.0106942.s004.doc]

**Supplementary Information**

**A Proteomic Approach to Investigating Gene Cluster Expression and Secondary Metabolite Functionality in *Aspergillus fumigatus*.**

Rebecca A. Owens, Stephen Hammel, Kevin J. Sheridan, Gary W. Jones and Sean Doyle*.

Department of Biology, National University of Ireland Maynooth, Maynooth, Co. Kildare, Ireland.

***Corresponding author**

Professor Sean Doyle,

Department of Biology,

National University of Ireland Maynooth,

Maynooth, Co. Kildare, Ireland.

Tel: +353-1-7083858; Fax: +353-1-7083845; E-mail: sean.doyle@nuim.ie

Web: http://biology.nuim.ie

**Keywords**

Fungal proteomics, gliotoxin, redox stress, NRPS, mycotoxin, LC-MS

**Table S3:** Proteins undergoing significant differential abundance^1^ in *A. fumigatus* ATCC26933 following exposure to gliotoxin and H_2_O_2_, separately or combined, relative to the solvent control. Protein identification was achieved by 2D-PAGE and LC-MS/MS.

| **Protein name** | **Gliotoxin v Control**^2^ | **H_2_O_2_**  **v Control**^2^ | **Co-addition v Control**^2^ | **Sequence coverage %** | **tM_r_ (Da)** | **CADRE ID. (AFUA_)** | **Spot No.** |
| --- | --- | --- | --- | --- | --- | --- | --- |
| Bifunctional purine biosynthetic protein Ade1 | ↓ 1.8 | - | - | 9 | 86419 | 6G04730 | 959 |
| Glycyl-tRNA synthetase | - | - | ↓ 1.9 | 4 | 79100 | 5G05920 | 305 |
| Hsp70 chaperone BiP/Kar2 | - | - | ↓ 1.7 | 24 | 73385 | 2G04620 | 988 |
| Methylenetetrahydrofolate reductase | - | - | ↓ 1.9 | 10 | 69279 | 2G11300 | 379 |
| Succinate dehydrogenase subunit Sdh1 | ↑ 1.7 | - | - | 18 | 71148 | 3G07810 | 960 |
| Thioredoxin reductase GliT | ↑ 5.1 | - | ↑ 4.8 | 16 | 36004 | 6G09740 | 738 |
| 14-3-3 family protein | - | ↑ 2.1 | - | 38 | 30104 | 6G06750 | 783 |
| Translation elongation factor EF2 subunit | ↑ 1.6 | ↑ 1.5 | ↑ 2.0 | 9 | 93198 | 2G13530 | 792 |
| Proliferating cell nuclear antigen (PCNA) | - | - | ↑ 5.9 | 8 | 24034 | 1G04900 | 980 |
| 14-3-3 family protein ArtA | - | ↑ 2.0 | ↑ 1.9 | 46 | 29102 | 2G03290 | 800 |
| Unknown function protein | ↑ 4.5 | - | ↑ 4.4 | 17 | 30152 | 2G11120 | 803 |
| Molecular chaperone and allergen Mod-E/Hsp90/Hsp1 | - | ↑ 2.0 | ↓ 1.5 | 8 | 80640 | 5G04170 | 966 |
| Proteasome component Pre8 | ↓ 1.8 | - | - | 27 | 30463 | 7G05870 | 853 |
| Unknown function protein | - | ↑ 2.3 | - | 10 | 63462 | 6G03460 | 964 |
| ATP synthase gamma chain, mitochondrial precursor, putative | - | ↑ 1.6 | ↑ 1.7 | 38 | 31547 | 1G03510 | 804 |
| Xanthine-guanine phosphoribosyl transferase Xpt1, putative | - | - | ↑ 1.8 | 28 | 19505 | 4G04550 | 992 |
| NADH-ubiquinone dehydrogenase 24 kDa subunit | - | - | ↑ 1.7 | 12 | 29771 | 2G09130 | 993 |
| Glutamine amidotransferase:cyclase | - | - | ↓ 1.9 | 27 | 60190 | 2G06230 | 968 |
| HAD superfamily hydrolase | - | - | ↑ 1.8 | 22 | 27360 | 5G08270 | 989 |

^1^*p*< 0.05; ^2^Fold increase (↑) or decrease (↓) of protein upon exposure of *A. fumigatus* ATCC26933 to gliotoxin alone, H_2_O_2_ alone or the co-addition, relative to control. CADRE ID., *A. fumigatus* gene annotation nomenclature according to Nierma*n et a*l. [22] and Mabe*y et a*l. [23]; tM_r_, theoretical molecular mass; Spot No, according to Figure 2; Co-addition: incubation with gliotoxin and H_2_O_2_ in combination.
