# Supplementary material for: A Proteomic Approach to Investigating Gene Cluster Expression and Secondary Metabolite Functionality in Aspergillus fumigatus
Source: PLoS One. 2014 Sep 8;9(9):e106942. doi: 10.1371/journal.pone.0106942 (PMC4157829; doi:10.1371/journal.pone.0106942)
Supplement: Table S4 — Proteins undergoing significant differential abundance1 in A. fumigatus ATCC26933 following exposure to a combination of gliotoxin and H2O2 (co-addition), relative to the control, gliotoxin alone or H2O2 alone. Protein identification was achieved by 2D-PAGE and LC-MS/MS. (DOC) [file pone.0106942.s005.doc]

**Supplementary Information**

**A Proteomic Approach to Investigating Gene Cluster Expression and Secondary Metabolite Functionality in *Aspergillus fumigatus*.**

Rebecca A. Owens, Stephen Hammel, Kevin J. Sheridan, Gary W. Jones and Sean Doyle*.

Department of Biology, National University of Ireland Maynooth, Maynooth, Co. Kildare, Ireland.

***Corresponding author**

Professor Sean Doyle,

Department of Biology,

National University of Ireland Maynooth,

Maynooth, Co. Kildare, Ireland.

Tel: +353-1-7083858; Fax: +353-1-7083845; E-mail: sean.doyle@nuim.ie

Web: http://biology.nuim.ie

**Keywords**

Fungal proteomics, gliotoxin, redox stress, NRPS, mycotoxin, LC-MS

**Table S4:** Proteins undergoing significant differential abundance^1^ in *A. fumigatus* ATCC26933 following exposure to a combination of gliotoxin and H_2_O_2_ (co-addition), relative to the control, gliotoxin alone or H_2_O_2_ alone. Protein identification was achieved by 2D-PAGE and LC-MS/MS.

| **Protein Name** | **Co-addition v Control^2^** | **Co-addition v Gliotoxin^2^** | **Co-addition v H_2_O_2_^2^** | **Sequence coverage %** | **tM_r_ (Da)** | **CADRE ID. (AFUA_)** | **Spot No.** |
| --- | --- | --- | --- | --- | --- | --- | --- |
| HAD superfamily hydrolase | ↑ 1.8 | ↑ 2.1 | ↑ 1.5 | 22 | 27360 | 5G08270 | 989 |
| Ran-specific GTPase-activating protein 1 | - | - | ↑ 1.8 | 13 | 27644 | 5G12180 | 850 |
| NADH-quinone oxidoreductase, 23 kDa subunit | - | - | ↑ 1.9 | 12 | 25752 | 1G06610 | 897 |
| Proliferating cell nuclear antigen (PCNA) | ↑ 5.9 | ↑ 7.4 | ↑ 2.4 | 8 | 24034 | 1G04900 | 980 |
| Thioredoxin reductase GliT | ↑ 4.8 | - | ↑ 3.5 | 16 | 36004 | 6G09740 | 738 |
| Unknown function protein | ↑ 4.4 | - | ↑ 4.9 | 17 | 30152 | 2G11120 | 803 |
| Glycyl-tRNA synthetase | ↓ 1.9 | - | ↓ 1.5 | 4 | 79100 | 5G05920 | 305 |
| Methylenetetrahydrofolate reductase | ↓ 1.9 | - | ↓ 1.6 | 10 | 69279 | 2G11300 | 379 |
| Oxidative stress protein Svf1 | - | - | ↓ 1.6 | 9 | 43421 | 5G11820 | 975 |
| Glutamine amidotransferase:cyclase | ↓ 1.9 | - | ↓ 1.7 | 27 | 60190 | 2G06230 | 968 |
| Class V chitinase | - | - | ↓ 1.8 | 24 | 43638 | 3G11280 | 662 |
| Molecular chaperone and allergen Mod-E/Hsp90/Hsp1 | ↓ 1.5 | - | ↓ 2.7 | 6 | 80640 | 5G04170 | 966 |
| Unknown function protein | - | - | ↓ 2.9 | 10 | 63462 | 6G03460 | 964 |
| Hsp70 chaperone BiP/Kar2 | ↓ 1.7 | ↓ 1.8 | - | 24 | 73385 | 2G04620 | 988 |
| F-actin capping protein alpha subunit | - | ↑ 1.7 | - | 18 | 30422 | 6G10060 | 790 |
| Translation elongation factor EF2 subunit | ↑ 2.0 | - | - | 9 | 93198 | 2G13530 | 792 |
| 14-3-3 family protein ArtA | ↑ 1.9 | - | - | 46 | 29102 | 2G03290 | 800 |
| Glucosamine-6-phosphate isomerase/6-phosphogluconolactonase family | - | ↑ 2.1 | - | 3 | 93269 | 1G02980 | 990 |
| Proteasome component Pre8 | - | ↑ 2.6 | - | 27 | 30463 | 7G05870 | 853 |
| Unknown function protein | - | ↑ 1.8 | - | 26 | 27921 | 3G00730 | 889 |
| ATP synthase gamma chain, mitochondrial precursor, putative | ↑ 1.7 | ↑ 1.6 | - | 38 | 31547 | 1G03510 | 804 |
| Xanthine-guanine phosphoribosyl transferase Xpt1, putative | ↑ 1.8 | ↑ 1.6 | - | 28 | 19505 | 4G04550 | 992 |
| NADH-ubiquinone dehydrogenase 24 kDa subunit | ↑ 1.7 | - | - | 12 | 29771 | 2G09130 | 993 |

^1^ *p* < 0.05; ^2^Fold increase (↑) or decrease (↓) of protein in the co-addition, relative to the solvent control, gliotoxin alone or H_2_O_2_ alone. CADRE ID., *A. fumigatus* gene annotation nomenclature according to Nierma*n et a*l. [22] and Mabe*y et a*l. [23]; tM_r_, theoretical molecular mass; Spot No, according to Figure 2; Co-addition: incubation with both gliotoxin and H_2_O_2_.
