# Supplementary material for: A Proteomic Approach to Investigating Gene Cluster Expression and Secondary Metabolite Functionality in Aspergillus fumigatus
Source: PLoS One. 2014 Sep 8;9(9):e106942. doi: 10.1371/journal.pone.0106942 (PMC4157829; doi:10.1371/journal.pone.0106942)
Supplement: Text S1 — Supplementary Discussion. (DOC) [file pone.0106942.s006.doc]

**Supplementary Information**

**A Proteomic Approach to Investigating Gene Cluster Expression and Secondary Metabolite Functionality in *Aspergillus fumigatus*.**

Rebecca A. Owens, Stephen Hammel, Kevin J. Sheridan, Gary W. Jones and Sean Doyle*.

Department of Biology, National University of Ireland Maynooth, Maynooth, Co. Kildare, Ireland.

***Corresponding author**

Professor Sean Doyle,

Department of Biology,

National University of Ireland Maynooth,

Maynooth, Co. Kildare, Ireland.

Tel: +353-1-7083858; Fax: +353-1-7083845; E-mail: sean.doyle@nuim.ie

Web: http://biology.nuim.ie

**Keywords**

Fungal proteomics, gliotoxin, redox stress, NRPS, mycotoxin, LC-MS

**Supplementary Discussion**

Functional classification of proteins identified by shotgun mass spectrometry revealed that categories describing: (i) protein synthesis, (ii) energy, (iii) protein with binding function or cofactor requirement and (iv) transcription were significantly represented in the data set. Identification of a large number of ribosomal proteins (*n* = 55) accounts for the high representation of protein synthesis and proteins with binding or cofactor requirement functional categories. Despite their cellular abundance [1], detection of ribosomal proteins by 2-D may be limited due the highly basic or acidic nature of these proteins and the relative low molecular mass of the subunits. Previous work [2] identified 11 ribosomal proteins during the compilation of the mycelial proteome reference map of *A. fumigatus* while no ribosomal proteins were detected in earlier proteome maps [3,4]. Of the ribosomal proteins identified by shotgun mass spectrometry, 49 exhibited a p*I* ≥ 8.59 and the remainder, described as acidic subunits, displayed a p*I* ≤ 5.74. All identified ribosomal proteins had a molecular mass ≤ 44 kDa, with 89.1 % having a M_r_ < 30 kDa. The functional category describing proteins involved in energy metabolism or transfer was also significantly over-represented from the sample set of proteins identified by shotgun mass spectrometry (*n* = 91; *p* = 4.22 x 10^-17^). The majority of the enzymes constituting the TCA cycle were detected, in addition to proteins involved in electron transport and energy generation by ATP synthases. This observation reflects the growth on glucose as a sole carbon source, whereby glycolysis provides a substrate for the TCA cycle which in turn generates energy via the electron transport chain. Enzymes were also identified that are involved in the pentose phosphate pathway, including 6-phosphogluconate dehydrogenase (AFUA_6G08050) which is responsible for the metabolism of 6-phospho-D-gluconate, a derivative of glucose, to D-ribulose 5-P [5].

A number of proteins (*n* = 23) were identified by shotgun proteomics with no functional annotation, based on FunCat, GO or KEGG annotation. BLAST2GO [6] analysis of these proteins revealed a number of putative functional annotations based on homology to more defined protein families. The protein encoded by AFUB_086030 was found to be associated with 14 GO terms using this method, and is a homologue of the hybrid PKS/NRPS from the pseurotin A biosynthetic cluster on Chromosome 8 (AFUA_8G00540; 99 % identity by BLAST). Individual identification of both AFUB_086030 and AFUA8G00540 was observed in this analysis, through detection of unique peptides that appear to differentiate between these homologues. These unique peptides arise from single amino acid substitutions and so may indicate subtle anomalies in the sequencing data from the two strains. A number of the proteins subjected to BLAST2GO analysis demonstrated no homology to any functionally defined protein families. These included the high expression lethality protein (AFUA_1G06580), cyanovirin-n family protein (AFUA_1G02290), the putative C6 transcription factor (AFUA_4G09250) and four unknown function proteins (UFPs) (AFUA_3G06460, AFUA_6G10450, AFUA_8G04890 and AFUA_8G05600). This study is the first report of the proteins encoded by AFUA_2G17000, AFUA_2G02490, AFUA_4G03322, AFUA_4G09250, AFUA_6G09920, AFUA_8G04890, with many others previously only identified at the transcript level [7–9].These proteins represent targets for future functional genomic or comparative proteomic projects to elucidate their role in fungi [10].

Comparative proteomics was implemented to characterise the effect of gliotoxin and H_2_O_2_ co-addition on *A. fumigatus*. In addition to the proteins discussed in the main body of the manuscript a number of other alterations were noted to the proteome. Increased abundance of NADH-quinone oxidoreductase (23 kDa subunit) was observed following exposure of *A. fumigatus* to a combination of gliotoxin and H_2_O_2_, relative to H_2_O_2_ alone (1.8 fold). Up-regulation of transcripts of this protein has previously been observed in *A. fumigatus* in response to the anti-malarial agent, artemisinin [11]. Artemisinins contain an endoperoxide bridge, essential for mediation of anti-malarial activity [12]. This protein forms part of complex I of the mitochondrially-located electron transport chain (ETC) and is involved in NADH oxidation [13]. Although complex I is actually a source of ROS in the electron transport chain [14], components of the ETC, including the alternative oxidase AoxA, are involved in fungal resistance to oxidative stress [15]. Additionally, generation of low-levels of ROS can elicit a signalling mechanism that promotes survival [16]. Alternatively, increased abundance of components of the ETC could be indicative of increased energy requirement due to recovery of growth in the co-exposure condition.

Abundance of Ran-specific GTPase-activating protein 1 (AFUA_5G12180) was also significantly induced by growth in gliotoxin combined with H_2_O_2_, relative to H_2_O_2_ alone (1.8 fold, *p* = 0.029). However, abundance of this protein did not demonstrate differential regulation relative to either the solvent control or gliotoxin treatment alone which may indicate that levels of this protein is down-regulated in the presence of H_2_O_2_ alone, and abundance returns to a basal level upon co-addition of gliotoxin. The Ran-specific GTPase-activating-protein 1 is involved in the up-regulation of protein degradation through ubiquitination and also functions in the regulation of nuclear import/export [17]. Depletion of the *S. cerevisiae* ortholog of this protein, Yrb1, correlates with cell-cycle arrest, underlining the importance of this protein during mitosis [17]. The level of Ran GTPase-activating protein 1 may account for the growth inhibition observed in the presence of H_2_O_2_ alone, relative to a combination of gliotoxin and H_2_O_2_ [18]. The subsequent increase in abundance of this protein upon incubation with gliotoxin and H_2_O_2_ in combination may influence recovery from H_2_O_2_-induced stress. Thus, the control of factors involved in cell-cycle regulation may be critical in the gliotoxin-mediated relief of H_2_O_2_-induced growth inhibition.

Abundance of the HAD superfamily hydrolase increased 1.5 fold in the presence of gliotoxin and H_2_O_2_, relative to H_2_O_2_ alone. Additionally, the HAD superfamily hydrolase exhibited increased abundance in the co-addition condition, relative to gliotoxin alone (2.1 fold), and the solvent control (1.8 fold) (Table 2), indicating that expression of this protein is affected by the combination of gliotoxin and H_2_O_2_ and not in singularity. A reduced abundance of the HAD superfamily hydrolase has been observed previously in *A. fumigatus* in response to hypoxic conditions [19]. While no defined metabolic role has been determined for the HAD superfamily hydrolase in *Aspergillus* species, an orthologous protein in *Penicillium brasiliensis* is necessary for adherence to host cells and the transcript was up-regulated in the transition from conidia to mycelial growth phases [20,21].

**References:**

[1] Warner JR. The economics of ribosome biosynthesis in yeast. Trends Biochem Sci 1999;24:437–40.

[2] Vödisch M, Albrecht D, Lessing F, Schmidt AD, Winkler R, Guthke R, et al. Two-dimensional proteome reference maps for the human pathogenic filamentous fungus *Aspergillus fumigatus*. Proteomics 2009;9:1407–15.

[3] Kniemeyer O, Lessing F, Scheibner O, Hertweck C, Brakhage AA. Optimisation of a 2-D gel electrophoresis protocol for the human-pathogenic fungus *Aspergillus fumigatus*. Curr Genet 2006;49:178–89.

[4] Carberry S, Neville CM, Kavanagh K, Doyle S. Analysis of major intracellular proteins of Aspergillus fumigatus by MALDI mass spectrometry: identification and characterisation of an elongation factor 1B protein with glutathione transferase activity. Biochem Biophys Res Commun 2006;341:1096–104.

[5] Maaheimo H, Fiaux J, Cakar P, Bailey JE, Sauer U, Szyperski T. Central carbon metabolism of *Saccharomyces cerevisiae* explored by biosyntheric fractional ^13^C labeling of common amino acids. Eur J Biochem / FEBSr 2001;268:2464–79.

[6] Conesa A, Götz S, García-Gómez JM, Terol J, Talón M, Robles M. Blast2GO: a universal tool for annotation, visualization and analysis in functional genomics research. Bioinformatics 2005;21:3674–6.

[7] Da Silva Ferreira ME, Malavazi I, Savoldi M, Brakhage AA, Goldman MHS, Kim HS, et al. Transcriptome analysis of Aspergillus fumigatus exposed to voriconazole. Curr Genet 2006;50:32–44.

[8] Sheppard DC, Doedt T, Chiang LY, Kim HS, Chen D, Nierman WC, et al. The *Aspergillus fumigatus* StuA Protein Governs the Up- Regulation of a Discrete Transcriptional Program during the Acquisition of Developmental Competence. Mol Biol Cell 2005;16:5866–79.

[9] Twumasi-Boateng K, Yu Y, Chen D, Gravelat FN, Nierman WC, Sheppard DC. Transcriptional profiling identifies a role for BrlA in the response to nitrogen depletion and for StuA in the regulation of secondary metabolite clusters in *Aspergillus fumigatus*. Eukaryot Cell 2009;8:104–15.

[10] Doyle S. Fungal proteomics: from identification to function. FEMS Microbiol Lett 2011;321:1–9.

[11] Gautam P, Upadhyay SK, Hassan W, Madan T, Sirdeshmukh R, Sundaram CS, et al. Transcriptomic and proteomic profile of *Aspergillus fumigatus* on exposure to artemisinin. Mycopathologia 2011;172:331–46.

[12] O’Neill PM, Barton VE, Ward S a. The molecular mechanism of action of artemisinin--the debate continues. Molecules 2010;15:1705–21.

[13] Lin S-J, Guarente L. Nicotinamide adenine dinucleotide, a metabolic regulator of transcription, longevity and disease. Curr Opin Cell Biol 2003;15:241–6.

[14] Voulgaris I, O’Donnell A, Harvey LM, McNeil B. Inactivating alternative NADH dehydrogenases: enhancing fungal bioprocesses by improving growth and biomass yield? Sci Rep 2012;2:322.

[15] Grahl N, Dinamarco TM, Willger SD, Goldman GH, Cramer RA. *Aspergillus fumigatus* mitochondrial electron transport chain mediates oxidative stress homeostasis, hypoxia responses and fungal pathogenesis. Mol Microbiol 2012;84:383–99.

[16] Trachootham D, Lu W, Ogasawara M a, Nilsa R-DV, Huang P. Redox regulation of cell survival. Antioxid Redox Signal 2008;10:1343–74.

[17] Baumer M, Kunzler M, Steigemann P, Braus GH, Irniger S. Yeast Ran-binding Protein Yrb1p Is Required for Efficient Proteolysis of Cell Cycle Regulatory Proteins Pds1p and Sic1p. J Biol Chem 2000;275:38929–37.

[18] Gallagher L, Owens RA, O’ Keeffe G, Dolan SK, Schrettl M, Kavanagh K, et al. The *Aspergillus fumigatus* Protein GliK Protects Against Oxidative Stress and is Essential for Gliotoxin Biosynthesis. Eukaryot Cell 2012;11:1226–38.

[19] Vödisch M, Scherlach K, Winkler R, Hertweck C, Braun H-P, Roth M, et al. Analysis of the *Aspergillus fumigatus* proteome reveals metabolic changes and the activation of the pseurotin A biosynthesis gene cluster in response to hypoxia. J Proteome Res 2011;10:2508–24.

[20] Hernández O, Almeida AJ, Gonzalez A, Garcia AM, Tamayo D, Cano LE, et al. A 32-Kilodalton Hydrolase Plays an Important Role in *Paracoccidioides brasiliensis* Adherence to Host Cells and Influences Pathogenicity. Infect Immun 2010;78:5280–6.

[21] Ndez OHÁ, Almeida AJ, Tamayo D, Torres I, Garcia ANAM, Pez ALÓ, et al. The hydrolase PbHAD32 participates in the adherence of *Paracoccidioides brasiliensis* conidia to epithelial lung cells. Med Mycol 2012;50:533–7.

[22] Nierman WC, Pain A, Anderson MJ, Wortman JR, Kim HS, Arroyo J, et al. Genomic sequence of the pathogenic and allergenic filamentous fungus *Aspergillus fumigatus*. Nature 2005;438:1151–6.

[23] Mabey JE, Anderson MJ, Giles PF, Miller CJ, Attwood TK, Paton NW, et al. CADRE: the Central Aspergillus Data REpository. Nucleic Acids Res 2004;32:D401–5.
